# Induction of Apoptosis in TNF-Treated L929 Cells in the Presence of Necrostatin-1

**DOI:** 10.3390/ijms17101678

**Published:** 2016-10-07

**Authors:** Hirofumi Sawai

**Affiliations:** Department of Internal Medicine, Osaka Dental University, Hirakata 573-1121, Japan; sawai@cc.osaka-dent.ac.jp; Tel.: +81-72-864-3079

**Keywords:** apoptosis, necroptosis, TNF, L929 cells, caspase-3, caspase-8, Z-VAD-fmk, Z-Asp-CH_2_-DCB

## Abstract

It has been shown that necroptosis—caspase-independent programmed necrotic cell death—can be induced by treatment with tumor necrosis factor (TNF) in the L929 murine fibrosarcoma cell line, even in the absence of a caspase inhibitor. Although it was reported that necrostatin-1—a specific inhibitor of necroptosis—inhibited TNF-induced necroptosis in L929 cells, it has not been elucidated whether the cells eventually die by apoptosis in the presence of necrostatin-1. In this paper, induction of apoptosis was demonstrated in TNF-treated L929 cells in the presence of necrostatin-1. Co-treatment with cycloheximide expedited apoptosis induction in necrostatin-1/TNF-treated L929 cells: typical apoptotic morphological changes, including membrane blebbing and nuclear fragmentation, induction of caspase-3 activity, proteolytic activation of caspases-3, -8, and -9, and cleavage of poly(ADP-ribose) polymerase (PARP) (a well-known substrate of caspase-3) were observed. Moreover, co-treatment with Z-VAD-fmk (a pan-caspase inhibitor) inhibited apoptosis by completely inhibiting caspases, resulting in a shift from apoptosis to necroptosis. In contrast, co-treatment with Z-Asp-CH_2_-DCB (a caspase inhibitor preferential to caspase-3) inhibited apoptosis without expediting necroptosis. These results indicate that apoptosis can be induced in TNF-treated L929 cells when the cells are protected from necroptosis, and support the notion that partial activation of caspase-8 in the presence of a caspase inhibitor preferential to caspase-3 suppresses both apoptosis and necroptosis.

## 1. Introduction

Apoptosis and necrosis are two major forms of cell death with distinguishing morphological features: the former is characterized by cell shrinkage, fragmented nuclei with condensed chromatin, and the formation of apoptotic bodies, whereas the latter is characterized by swelling of the cells and organelles and loss of plasma membrane integrity [1,2,3]. The molecular mechanism of apoptosis was investigated in detail in the 1990s, and it has been established that caspases play crucial roles in the induction of apoptotic cell death [4]. In contrast to post-apoptotic secondary necrosis, caspase-independent programmed necrotic cell death—termed necroptosis—has been elucidated, owing to the identification of necrostatins—specific inhibitors of necroptosis—in the 2000s [5]. Thereafter, the molecular mechanism of necroptosis has been investigated in detail: receptor-interacting protein 1 (RIP1), whose kinase activity is inhibited by necrostatins [6], RIP3, and mixed lineage kinase domain-like protein (MLKL) are critically involved in necroptotic cell death [7,8,9]. Although apoptosis has been reported to be involved in various pathological conditions, recent studies have suggested that necroptosis plays a critical role in several disorders, including myocardial infarction, stroke, ischemia-reperfusion injury, and inflammatory bowel disease [10].

Whereas necroptosis has been induced in the presence of caspase inhibitors in many types of cells, it has been reported that necroptosis can be induced by the treatment with tumor necrosis factor (TNF) in the L929 murine fibrosarcoma cell line, even in the absence of caspase inhibitors [11,12]. Although it was reported that necrostatin-1 inhibited TNF-induced necroptosis in L929 cells [5,8], it has not been elucidated whether the cells treated with TNF in the presence of necrostatin-1 eventually die by apoptosis. In this paper, induction of apoptosis was demonstrated in TNF-treated L929 cells in the presence of necrostatin-1.

## 2. Results

### 2.1. Induction of Necroptosis in TNF-Treated L929 Cells

**Figure 1:**
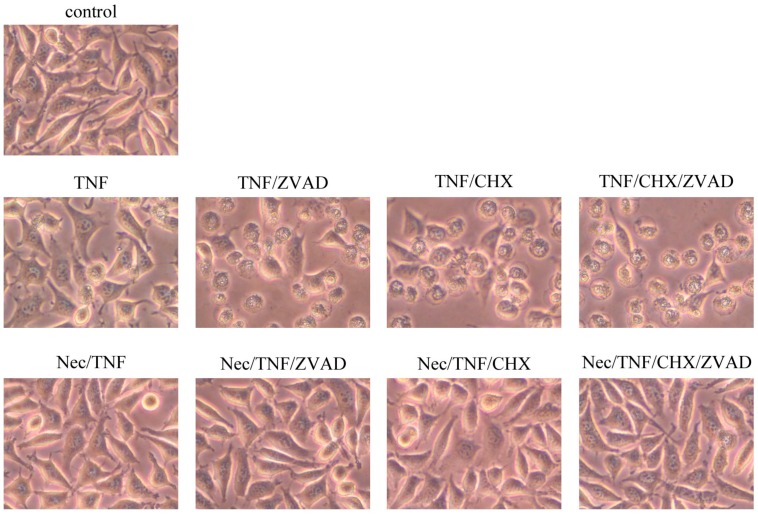
Induction of necroptosis in tumor necrosis factor (TNF)-treated L929 cells. L929 cells were treated with 10 ng/mL TNF, 10 μM Z-VAD-fmk (ZVAD), 10 μg/mL cycloheximide (CHX), or 10 μM necrostatin-1 (Nec) for 3 h. Photos of phase-contrast microscopy (original magnification 400×) are shown.

As reported [11,12], necrotic cell death was induced in L929 cells by treatment with TNF. Morphologically, swelling of the cells without nuclear fragmentation was observed in TNF-treated L929 cells (Figure 1). TNF-induced necrotic cell death was expedited in the presence of Z-VAD-fmk (ZVAD), a pan-caspase inhibitor, and most of the cells became necrotic within 3 h of treatment with TNF/ZVAD. TNF-induced necrosis was also expedited in the presence of cycloheximide (CHX), a protein synthesis inhibitor.

**Figure 2:**
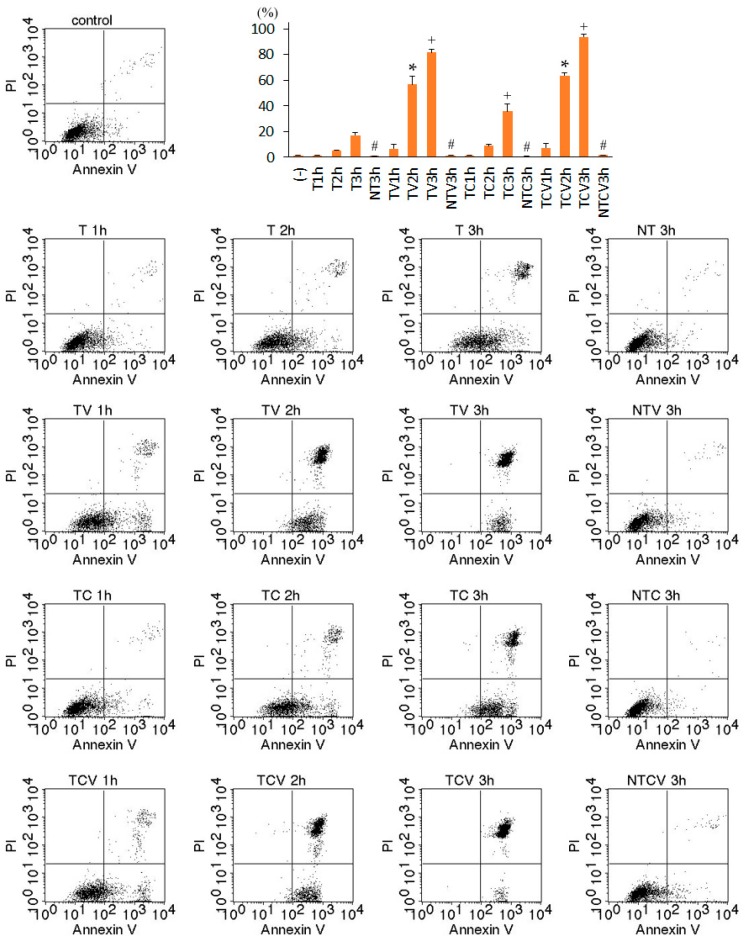
Dual staining of L929 cells with Annexin V (AnV) and propidium iodide (PI). L929 cells were treated with 10 ng/mL TNF (T), 10 μM Z-VAD-fmk (V), 10 μg/mL cycloheximide (C), or 10 μM necrostatin-1 (N) for the indicated times. AnV^+^/PI^−^: early necroptosis; AnV^+^/PI^+^: necrosis (late necroptosis). The graph shows the average of three independent experiments for AnV^+^/PI^+^ cells. * *p* < 0.05 compared to T2h; ^+^ *p* < 0.05 compared to T3h; ^#^ *p* < 0.05 compared to 3 h without necrostatin-1.

Next, necrotic cell death of TNF-treated L929 cells was assessed by dual staining with Annexin V (AnV) and propidium iodide (PI) (Figure 2). An increase in AnV^+^/PI^−^ cells and AnV^+^/PI^+^ (necrotic) cells was observed in TNF-treated L929 cells within 3 h. Co-treatment with either ZVAD or CHX significantly expedited the appearance of AnV^+^/PI^−^, followed by AnV^+^/PI^+^ cells. As previously reported [5,13,14], necrosis of TNF-treated L929 cells was completely inhibited in the presence of necrostatin-1, an inhibitor of RIP1 kinase activity (Figure 1 and Figure 2). Furthermore, TNF/ZVAD-induced or TNF/CHX-induced L929 cell death was also almost completely inhibited by necrostatin-1. These results indicated that TNF-induced L929 cell death was necroptosis.

### 2.2. Induction of Apoptosis in TNF-Treated L929 Cells in the Presence of Necrostatin-1

**Figure 3:**
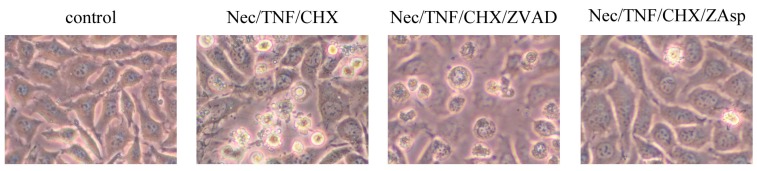
Induction of apoptosis in TNF-treated L929 cells in the presence of necrostatin-1 (phase-contrast microscopy, original magnification 400×). L929 cells were treated with 10 μM necrostatin-1 (Nec), 10 ng/mL TNF, 10 µg/mL cycloheximide (CHX) with or without 10 μM Z-VAD-fmk (ZVAD) or 100 μM Z-Asp-CH_2_-DCB (ZAsp) for 5 h.

Whether TNF-treated L929 cells eventually die by apoptosis in the presence of necrostatin-1 was investigated. To expedite cell death, L929 cells were treated with TNF and CHX in these experiments, since treatment of L929 cells with TNF in the presence of necrostatin-1 did not induce cell death, even after 24 h. Although TNF/CHX-induced necrotic cell death was almost completely inhibited in the presence of necrostatin-1 within 3 h (Figure 1 and Figure 2), typical apoptotic changes such as cell shrinkage and membrane blebbing were observed at 4 h or later (Figure 3).

**Figure 4:**
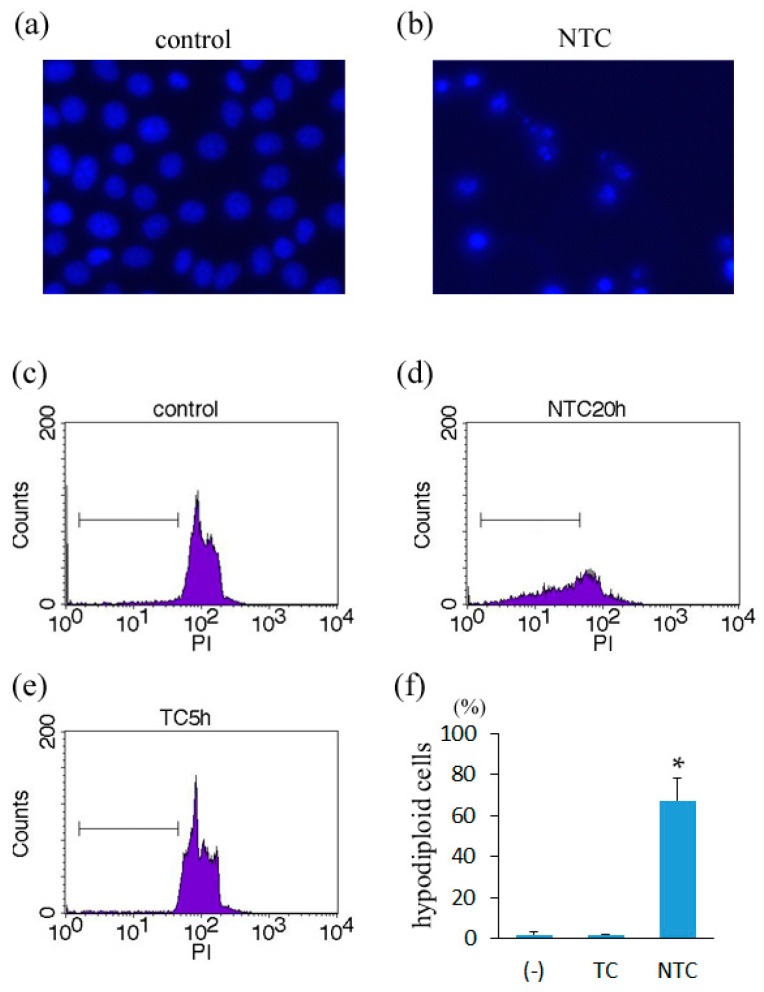
Induction of apoptosis in Nec/TNF/CHX-treated L929 cells (nuclear fragmentation). (**a**,**b**) Hoechst 33342 staining (original magnification 200×). L929 cells were treated with 10 μM necrostatin-1 (N), 10 ng/mL TNF (T), and 10 μg/mL cycloheximide (C) for 20 h; (**c**–**f**) Cellular DNA content was analyzed using flow cytometry. L929 cells were treated with (**d**) 10 μM necrostatin-1 (N), 10 ng/mL TNF (T), and 10 μg/mL cycloheximide (C) for 20 h, or (**e**) 10 ng/mL TNF (T) and 10 μg/mL cycloheximide (C) for 5 h. The graph shows the average of three independent experiments. The bars indicate one standard deviation. * *p* < 0.01 compared with non-treated control. PI, propidium iodide.

To confirm that apoptosis was induced in necrostatin-1/TNF/CHX-treated cells, the cells were stained with Hoechst 33342 to examine nuclear morphology. Control (non-treated) cells showed normal nuclear morphology (Figure 4a), whereas cells treated with necrostatin-1/TNF/CHX for 20 h showed condensed chromatin and nuclear fragmentation (Figure 4b). Furthermore, analysis of cellular DNA content demonstrated that most necrostatin-1/TNF/CHX-treated cells became hypodiploid, indicating apoptotic nuclear fragmentation (Figure 4c,d,f). These results confirm that apoptosis is induced in L929 cells by treatment with necrostatin-1/TNF/CHX. In contrast, no increase in hypodiploid cells was detected in L929 cells treated with TNF/CHX for 5 h (Figure 4e,f), with TNF alone, or with necrostatin-1/TNF/CHX for 5 h.

**Figure 5:**
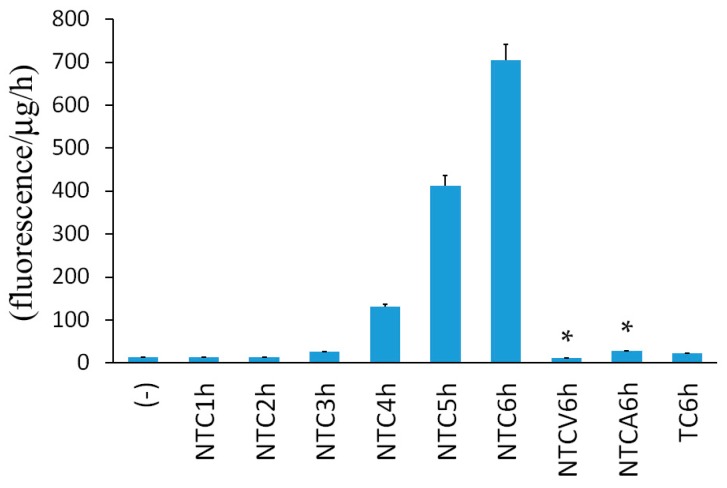
Induction of caspase-3 activity in Nec/TNF/CHX-treated L929 cells. L929 cells were treated with 10 μM necrostatin-1 (N), 10 ng/mL TNF (T), and 10 μg/mL cycloheximide (C) with or without 10 μM Z-VAD-fmk (V) or 100 μM Z-Asp-CH_2_-DCB (A) for the indicated times. The data shown are the average of a triplicate experiment. The bars indicate one standard deviation. * *p* < 0.01 compared with NTC6h. Similar results were obtained in three independent experiments.

Furthermore, changes in caspase-3 activity were examined. As shown in Figure 5, a slight increase in caspase-3 activity was detected at 4 h in the cells treated with necrostatin-1/TNF/CHX, and thereafter caspase-3 activity was increased in a time-dependent manner. In contrast, caspase-3 activity was not apparently increased in TNF/CHX-treated L929 cells in the absence of necrostatin-1.

**Figure 6:**
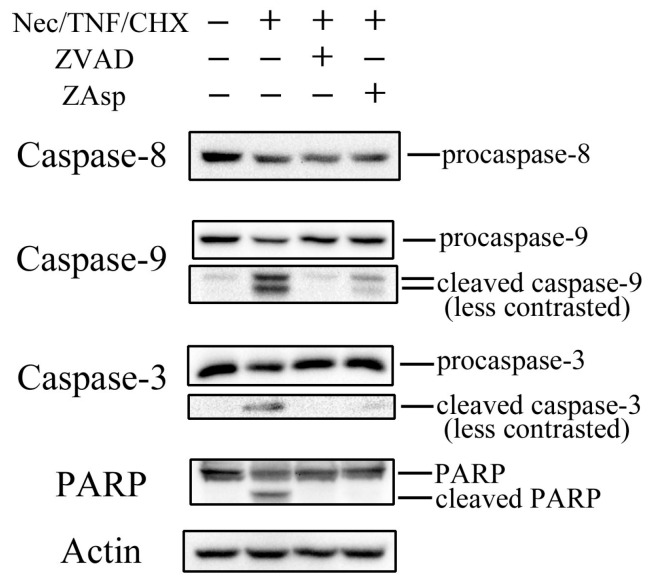
Western blot analysis of proteolytic activation of Caspases. L929 cells were treated with 10 μM necrostatin-1 (Nec), 10 ng/mL TNF, and 10 μg/mL cycloheximide (CHX) with or without 10 μM Z-VAD-fmk (ZVAD) or 100 μM Z-Asp-CH_2_-DCB (ZAsp) for 5 h, and Western blot analysis of Caspases-3, -8, -9, and Poly(ADP-Ribose) Polymerase (PARP) was performed. The images shown are representative of three independent experiments with similar results.

Moreover, activation of caspases was assessed by Western blot analysis (Figure 6). When the cells were treated with necrostatin-1/TNF/CHX for 5 h, reduction of procaspases-3, -8, and -9 with concomitant appearance of cleaved caspases-3 and -9 was observed. The level of PARP—a well-known substrate of caspase-3—was also reduced, with the concomitant appearance of cleaved PARP in necrostatin-1/TNF/CHX-treated cells, indicating the activation of caspase-3.

### 2.3. Differential Effects of Caspase Inhibitors on Necrostatin-1/TNF-Treated L929 Cells

**Figure 7:**
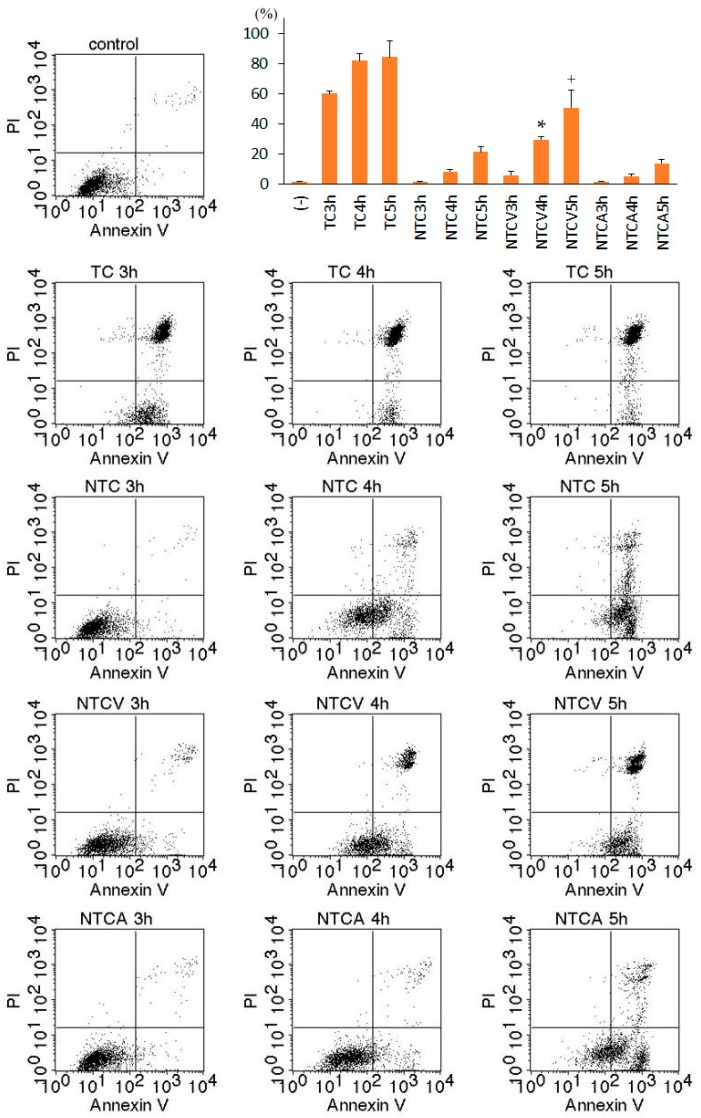
Induction of apoptosis in TNF-treated L929 cells in the presence of necrostatin-1 (dual staining with Annexin V and propidium iodide). L929 cells were treated with 10 μM necrostatin-1 (N), 10 ng/mL TNF (T), 10 μg/mL cycloheximide (C) with or without 10 μM Z-VAD-fmk (V) or 100 μM Z-Asp-CH_2_-DCB (A) for the indicated times. AnV^+^/PI^−^: early apoptosis or early necroptosis; AnV^+^/PI^+^: necrosis (late apoptosis or late necroptosis). The graph shows the average of three independent experiments for AnV^+^/PI^+^ cells. * *p* < 0.05 compared to NTC4h; ^+^ *p* < 0.05 compared to NTC5h.

Dual staining with AnV and PI demonstrated the appearance of AnV^+^/PI^−^ cells followed by AnV^+^/PI^+^ cells in necrostatin-1/TNF/CHX-treated cells at 4 h or later (Figure 7), suggesting that these AnV^+^/PI^−^ and AnV^+^/PI^+^ cells were early and late apoptotic, respectively. It was previously reported that ZVAD inhibited apoptosis but induced necroptosis, whereas Z-Asp-CH_2_-DCB (ZAsp)—a caspase inhibitor preferential to caspase-3—suppressed apoptosis without inducing necroptosis in TNF-treated U937 human monocytic leukemia cells [15]. As shown in Figure 3, necrotic cells showing cell swelling without apoptotic morphology were observed in L929 cells treated with necrostatin-1/TNF/CHX in the presence of ZVAD. Moreover, co-treatment with ZVAD expedited the appearance of necrotic AnV^+^/PI^+^ cells in necrostatin-1/TNF/CHX-treated cells (Figure 7), suggesting the induction of necroptosis due to the inhibition of apoptosis by ZVAD. In contrast, co-treatment with ZAsp at least partially inhibited apoptosis without expediting necrotic cell death (Figure 3), and inhibited the appearance of both AnV^+^/PI^−^ and AnV^+^/PI^+^ cells in necrostatin-1/TNF/CHX-treated cells (Figure 7). These results indicated that ZAsp inhibited apoptosis without expediting necroptosis, which is consistent with the previous report [15]. As shown in Figure 6, proteolytic activation of caspases-9 and -3 (which depends on activated caspase-8 in the death-receptor pathway [4]) was completely inhibited by ZVAD, whereas cleaved caspases-9 and -3 were slightly detected in the presence of ZAsp. These results are also consistent with the previous report [15], suggesting that partial activation of caspase-8 in the presence of ZAsp may suppress the induction of necroptosis.

## 3. Discussion

TNF induces caspase-dependent apoptosis in many types of cells, whereas it induces caspase-independent necroptosis in the presence of caspase inhibitors such as Z-VAD-fmk. In contrast, TNF induces necroptosis even in the absence of caspase inhibitors in L929 cells. Although it was reported that necrostatin-1 (an inhibitor of necroptosis) inhibited TNF-induced necroptosis in L929 cells, it remained to be determined whether L929 cells treated with necrostain-1/TNF eventually die by apoptosis. In this paper, it is demonstrated that apoptosis can be induced in TNF-treated L929 cells in the presence of necrostatin-1. Necrostatin-1 has been identified as a specific inhibitor of RIP1 kinase activity [6]. It has been demonstrated that RIP1 is a pleiotropic adaptor protein mediating the activation of NF-κB and mitogen-activated protein kinase (MAPK), apoptosis, and necroptosis [16]. It was reported that RIP1 depletion blocked TNF-induced necroptosis in the presence of ZVAD in L929 cells [8,14], although it did not protect L929 cells from TNF-induced cell death in the absence of ZVAD due to a switch from necroptosis to apoptosis [14]. Necrostatin-1 induced a shift from necroptosis to apoptosis in TNF-treated L929 cells in this study, suggesting that the inhibition of RIP1 kinase activity, but not the depletion of RIP1, is sufficient to induce a switch from necroptosis to apoptosis. A recent report showed that depletion of RIP3 or MLKL induced a switch from necroptosis to apoptosis in TNF-treated L929 cells [17], consistent with the notion that RIP1 depletion is not necessary for the shift from necroptosis to apoptosis.

It was previously reported that co-treatment with Z-Asp-CH_2_-DCB (ZAsp, a caspase inhibitor preferential to caspase-3) inhibited TNF-induced apoptosis without expediting necroptosis in U937 cells, while co-treatment with ZVAD (a pan-caspase inhibitor) induced necroptosis by inhibiting apoptosis [15]. In this paper, ZAsp inhibited necrostatin-1/TNF/CHX-induced apoptosis without expediting necroptosis in 929 cells, while ZVAD induced a switch from apoptosis to necrosis in necrostatin-1/TNF/CHX-treated cells, which is consistent with the previous report [15]. These results support the notion that partial activation of caspase-8 in the presence of ZAsp may suppress necroptosis, possibly due to the cleavage of proteins involved in necroptosis, such as RIP1 [18], RIP3 [19], and CYLD [20], by activated caspase-8.

It is generally thought that apoptosis is tolerogenic (non-immunogenic), whereas necroptosis is immunogenic, although apoptosis can be immunogenic depending on the stimuli [21,22]. Therefore, a shift from apoptosis to necroptosis or vice versa may greatly affect immunological responses to dead cells. For example, a switch from apoptosis to necroptosis by caspase inhibitors such as ZVAD might augment immunological responses against malignant tumor cells treated with anticancer drugs, and a shift from necroptosis to apoptosis by necrostatins might reduce detrimental immunological responses against myocardial infarction and stroke. Further studies are required to elucidate the immunological effect of pharmacological manipulation of apoptosis and necroptosis on various pathological conditions.

In conclusion, apoptosis can be induced in TNF-treated L929 cells when the cells are protected from necroptosis by necrostatin-1. It is recognized that TNF induces both apoptotic and necroptotic signaling. In many cells treated with TNF, apoptotic signaling occurs more rapidly than necroptotic signaling. Moreover, activated caspase-8 in apoptotic signaling cleaves proteins involved in necroptotic signaling, such as RIP1, RIP3, and CYLD, resulting in the inhibition of necroptosis. In contrast, necroptotic signaling occurs more rapidly than apoptotic signaling in TNF-treated L929 cells. Therefore, the inhibition of necroptotic signaling by necrostatin-1 induces apoptosis at a later time point in L929 cells. The mechanism by which necroptotic signaling occurs more rapidly than apoptotic signaling in TNF-treated L929 cells remains to be determined.

## 4. Materials and Methods

### 4.1. Materials

L929 mouse fibrosarcoma cells were purchased from JCRB Cell Bank (Ibaraki, Japan). Necrostatin-1, cycloheximide, propidium iodide (PI) solution, and Hoechst 33342 were purchased from Sigma-Aldrich (St. Louis, MO, USA). Murine recombinant TNF-α was purchased from Peprotec. Z-VAD-fmk, Z-Asp-CH_2_-DCB, and Ac-Asp-Glu-Val-Asp(DEVD)-MCA was purchased from Peptide Institute (Ibaraki, Japan). Anti-mouse Caspase-3 antibody (clone 46) were purchased from BD Biosciences (San Jose, CA, USA). Anti-mouse Caspase-8 (#4927) and anti-PARP (#9542) antibodies were purchased from Cell Signaling Technology (Danvers, MA, USA). Anti-Caspase-9 antibody (clone 5B4) and fluorescein isocyothianate (FITC)-conjugated Annexin V were purchased from MBL (Nagoya, Japan). Horseradish peroxidase (HRP)-conjugated anti-Actin and secondary antibodies were purchased from Santa Cruz Biotechnology (Dallas, TX, USA).

### 4.2. Cell Culture

L929 cells were maintained in Dulbecco’s modified Eagle medium (D-MEM) (Wako, Osaka, Japan) supplemented with 10% heat-inactivated fetal bovine serum and penicillin (100 U/mL)/streptomycin (100 μg/mL) (Nacalai Tesque, Kyoto, Japan) in a humidified 5% CO_2_ incubator at 37 °C. For trypsinization, trypsin (0.25%)/EDTA (1 mM) solution (Nacalai Tesque, Kyoto, Japan) was used.

### 4.3. Dual Staining with Annexin V and Propidium Iodide

The cells were trypsinized and inoculated in non-treated 24 well plates (0.5 mL in each well). After 1 h, the cells were treated with 10 ng/mL TNF in the presence or absence of 10 μM necrostatin-1, 10 ng/mL cycloheximide, 10 μM Z-VAD-fmk, or 100 μM Z-Asp-CH_2_-DCB for the indicated times. Then, the cells were collected by pipetting only (without trypsinization). After centrifugation, the cells were incubated in 100 μL binding buffer (10 mM Hepes/KOH (pH 7.4), 140 mM NaCl, and 2.5 mM CaCl_2_) containing 5 μL FITC-conjugated Annexin V and 2.5 μg/mL PI for 30 min at 4 °C. The cells were analyzed using a FACS Calibur flow cytometer and Cell Quest software (BD Biosciences, San Jose, CA, USA). As was previously reported [23,24], not only early apoptotic cells but also early necroptotic cells are Annexin V^+^/PI^−^ before becoming PI^+^, which can be discriminated by co-treatment with necrostatin-1: the former are only slightly affected, whereas the latter are almost completely inhibited by necrostatin-1. Apoptosis and necroptosis are primarily judged from morphology. Annexin V/PI double staining is used to detect early apoptotic or early necroptotic cells, both of which are Annexin V^+^/PI^−^.

### 4.4. Hoechst 33342 Staining

The cells were fixed with 4% paraformaldehyde in PBS at room temperature for 5 min. Then the cells were stained with 10 μM Hoechst 33342 for 5 min, and nuclear morphology was observed under fluorescent microscope, BZ-9000 (Keyence, Osaka, Japan).

### 4.5. Analysis of Cellular DNA Content

The cells were trypsinized and inoculated in non-treated 24 well plates (0.5 mL in each well). After 1 h, the cells were treated with 10 μM necrostatin-1, 10 ng/mL TNF, and 10 ng/mL cycloheximide overnight. The cells were collected by pipetting only, and fixed with 1% paraformaldehyde in phosphate-buffered saline (PBS) containing 0.5% saponin for 5 min at 4 °C. After centrifugation, the cells were incubated in buffer containing 5 μg/mL propidium iodide and 1 mg/mL ribonuclease A (Nacalai Tesque, Kyoto, Japan) for 10 min at 4 °C. Then, the cells were analyzed using FACS Calibur flow cytometer and Cell Quest software.

### 4.6. Measurement of Caspase-3 Activity

L929 cells were trypsinized and inoculated in treated 12-well plates (1 mL in each well). After overnight incubation, the cells were treated with 10 ng/mL TNF in the presence or absence of 10 μM necrostatin-1, 10 ng/mL cycloheximide, 10 μM Z-VAD-fmk, or 100 μM Z-Asp-CH_2_-DCB for the indicated times. Then, the cells were scraped from the plates and centrifuged. After washing with phosphate-buffered saline (PBS) three times, the cells were lysed in buffer containing 10 mM Hepes/KOH (pH 7.4), 1% Triton X-100, 5 mM EDTA, and 1 mM phenylmethylsulfonyl fluoride (PMSF). After centrifugation at 10,000× *g* for 10 min, 5 μL of the supernatant was incubated with 100 μL reaction mixture containing 10 mM Hepes/KOH (pH 7.4), 0.1% 3-[(3-cholamidepropyl)dimethylammonio]propanesulfonate (CHAPS), 10% sucrose, 1 mM dithiothreitol, and 10 μM Ac-DEVD-MCA in black 96-well plates for 1 h at 37 °C. Fluorescence was measured using SpectraMax M5 (Molecular Devices, Sunnyvale, CA, USA) with excitation at 355 nm and emission at 460 nm. The protein concentration of the supernatant was analyzed using Bio-Rad Protein Assay, and the value of fluorescence was divided by the protein amount of the supernatant.

### 4.7. Western Blot Analysis

The cells were trypsinized and inoculated in treated 6-well plates (2 mL in each well). After overnight incubation, the cells were treated with 10 ng/mL TNF and 10 ng/mL cycloheximide in the presence or absence of 10 μM necrostatin-1, 10 μM Z-VAD-fmk, or 100 μM Z-Asp-CH_2_-DCB for the indicated times. Then, the cells were scraped from the plates and centrifuged. After washing with PBS, the cells were lysed in buffer containing 25 mM Tris/HCl (pH 7.4), 1% Triton X-100, 5 mM EDTA, and 1% protease inhibitor cocktail. After centrifugation at 400× *g* for 5 min, the supernatant was collected. Twenty μg protein was applied to each well, was separated in SDS-PAGE, and was transferred onto Immobilon polyvinylidene difluoride (PVDF) membrane (Merck Millipore, Darmstadt, Germany). After preincubation in PBS containing 0.1% Tween 20 (PBS-T) plus 10% Blocking One (Nacalai Tesque, Japan) for 30 min, the membrane was incubated with anti-caspases-3, -8, -9, or anti-PARP for 2 h. After washing in PBS-T for 15 and 5 min, the membrane was incubated with respective secondary antibody conjugated with HRP for 1 h. Then the membrane was washed in PBS-T for 5 min three times, and incubated in Supersignal West Pico chemiluminescent substrate (ThermoFischer Scientific, Yokohama, Japan) according to the manufacturer’s protocol. The chemiluminescent image was obtained using ChemiDoc (Bio-Rad, Hercules, CA, USA). The membrane was reprobed with HRP-conjugated anti-Actin antibody to confirm equal protein loading.

### 4.8. Statistical Analysis

For statistical analysis, one-way ANOVA with a post-hoc test (Tukey) was performed using SPSS Statistics 21.0 (IBM, Armonk, NY, USA).
